# Sustainable pig diets: partial grain replacement with former food products and its impact on meat quality

**DOI:** 10.1093/jas/skae070

**Published:** 2024-03-15

**Authors:** Marco Tretola, Sharon Mazzoleni, Paolo Silacci, Sébastien Dubois, Cristina Proserpio, Ella Pagliarini, Cristian E M Bernardi, Luciano Pinotti, Giuseppe Bee

**Affiliations:** Agroscope, Posieux 1725, Switzerland; Department of Veterinary Medicine and Animal Science, DIVAS, University of Milan, Lodi 26900, Italy; Department of Veterinary Medicine and Animal Science, DIVAS, University of Milan, Lodi 26900, Italy; Agroscope, Posieux 1725, Switzerland; Agroscope, Posieux 1725, Switzerland; Sensory & Consumer Science Lab (SCS_Lab), Department of Food, Environmental and Nutritional Sciences, DeFENS, University of Milan, Milano 20133, Italy; Sensory & Consumer Science Lab (SCS_Lab), Department of Food, Environmental and Nutritional Sciences, DeFENS, University of Milan, Milano 20133, Italy; Department of Veterinary Medicine and Animal Science, DIVAS, University of Milan, Lodi 26900, Italy; Department of Veterinary Medicine and Animal Science, DIVAS, University of Milan, Lodi 26900, Italy; CRC I-WE, Coordinating Research Centre: Innovation for Well-Being and Environment, University of Milan, Milan 20134, Italy; Agroscope, Posieux 1725, Switzerland

**Keywords:** former food products, meat quality, sustainable diets, sensory properties

## Abstract

This study investigated the effects of salty and sugary former foodstuff products (FFPs) on the quality traits and meat composition of 36 male castrated pigs (Swiss Large White breed) as well as sensory characteristics of the loins. The animals were fed three different diets for both the growing (G) and finishing (F) phases: (1) a standard diet (ST), 0% FFPs; (2) a diet with 30% of sugary FFPs (e.g., chocolate, biscuits, cakes) as a replacement for traditional ingredients (SU); and (3) a diet with 30% of salty FFPs (e.g., bread, pasta, and breadsticks) as a replacement for traditional ingredients (SA). For a comprehensive assessment of meat quality, protein and fat content in the LD were analyzed. AA and FA profile were determined both in the LD and backfat. Meat quality traits such as pH and temperature, thawing, cooking and drip losses, and shear force have been evaluated. Then, pork loins have been assessed for sensory attributes by a trained sensory panel. The SA diet decreased 20:5 n-3 levels (*P* < 0.001) in the muscle and 22:5 n-3 levels (*P* < 0.05) in both muscle and backfat but increased (*P* < 0.05) the ratio of mono-unsaturated to saturated fatty acids compared to the ST group. Both the SU and SA diets elevated (*P* < 0.001) the n-6:n-3 fatty acids ratio compared to the ST diet. Dietary treatments did not affect other meat quality traits. Regarding sensory attributes, the loin from pigs fed with SU and SA diets were sweeter (*P* < 0.001). Loins of SA pigs were more tender (*P* < 0.001), had a more intense pork aroma (*P* < 0.001) and had more flavor (*P* < 0.01) compared to ST loins. Overall, the use of FFPs affected the fatty acid profile of pork while improving the sensory quality of the loins, with no negative effects observed on the technological and nutritional quality of the meat.

## Teaser

Reintroducing food losses, also known as former food products into pigs’ diets contributes to the sustainability of livestock production. Former food products as alternative feed ingredients do not compromise technological and nutritional pork quality or consumer perception.

## Graphical Abstract

**Graphical Abstract:**
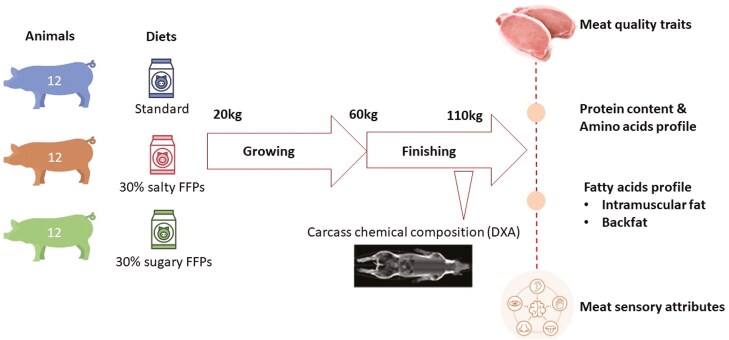

## Introduction

To manufacture feed, a variety of ingredients are available, yet it’s crucial to acknowledge that feed production carries an environmental footprint. Therefore, there’s a pressing need to curtail the consumption of natural resources by enhancing their reutilization and adopting “circular” feed sources. In this context, ex-food, also known as former food products (FFPs), emerges as a compelling solution. FFPs offer a means to transform losses from the food industry into ingredients for the feed sector, thereby ensuring the retention of nutrients within the food chain. This approach not only mitigates waste generation but also contributes to sustainability efforts within the agricultural and food industries. FFPs are food surplus originating from the confectionery and bakery food industries, comprise of ultra-processed food rejected for human consumption due to errors in the color, shape, flavor, or labelling of the product, logistical problems, or damaged packaging. These FFPs encompass salty foodstuffs, such as pasta, bread, and salty snacks, and sugary treats, such as cocoa-based products, candies, biscuits, and cereal bars (Pinotti et al., 2021). In general, FFPs consist of a blend of the above-mentioned sources of salty and sugary food, supplied by different manufactures. The mixture of starting ingredients characterized by different nutritional composition can lead to a significant variability, however FFPs producers are able to predict the range of variation in the analysis between different sources of product and between the same source and different loads, owing to years of experience in the analysis of incoming products. Therefore, FFPs producers know how to predict the concentration of nutrients, obtaining a final product which is consistent and standardized (Tretola et al., 2019a). Distinct from food waste from restaurants, retail chains, or households, FFPs are considered microbiologically safe, and they undergo a different legislation compared to food waste (European Commission, 2018). In accordance with European Commission feed legislation, food waste can be intended for recycling (e.g., anaerobic digestion, composing) or can be recovered by incineration to produce energy, but it cannot be transformed into feed and re-enter the food chain (European Commission, 2018). Conversely, FFPs can be “redistributed to people” and “transformed into animal feed”, since they are not considered as food scraps. Contrariwise, recycling and converting food waste into animal feed, after undergoing thermal processes, has been promoted in many non-European countries including Japan, South Korea, China, Taiwan, and United States (Rajeh et al., 2021; James et al., 2022). The FFPs are a valid source of monosaccharides, lipids and highly digestible starch, since they are pre-cooked during the industrial processing. These characteristics suggest that FFPs are quite similar to common cereals, however they contain higher levels of fats, and they undergo to heat treatments, which make them suitable for young animals (Ottoboni et al., 2019). Tretola et al., 2019a and Luciano et al. (2021; 2022) reported that FFPs can be used as ingredients in piglet diets, showing no adverse effects on growth performance, diet digestibility, gut microbiota, or metabolic profile. These encouraging results showed the potential of utilizing FFPs instead of cereals in swine diets to keep nutrients and reduce food losses within the agri-food chain and consequently mitigate the competition for natural resources in the production of livestock feed and human food (Pinotti et al., 2023a). Although food surplus represents a highly available biomass to be used as animal feed (about 5 million tons of FFPs produced/per year in the EU), only 3% of such biomass is actually reused and the remaining part is mainly destined to landfill or incineration, causing an environmental sustainability issue. This scenario is even more important at global level where if all non-usable food materials were used as feed for animals, these items may contribute up to 15% of total feed used for livestock (Sandström et al., 2022). Among livestock species, pigs, owing their omnivorous nature, can convert FFPs which are no more suitable for human consumption, into high quality animal proteins (Pinotti et al., 2023a). While sustainability gains precedence, maintaining meat quality remains paramount in the pork industry. Consumers demand high-quality pork products with excellent technological, nutritional, and sensory attributes (Liu et al., 2022; Pinotti et al., 2023b). Technological pork quality traits, nutritional value, and sensory attributes may be affected before and after slaughter, as well as at slaughter, by multiple interacting factors, such as feeding strategies (Lebret and Čandek-Potokar, 2022). It is known that the amount of intramuscular fat (IMF) has a certain impact on the quality and sensory properties of pork (Hoa et al., 2021). For instance, the increase in the lipid content (highly marbled pork) positively influences the sensory attributes of meat such as texture, tenderness, flavor, and juiciness (Cannata et al., 2010). Conversely, a reduction of the lipid content is linked to a decreased water-holding capacity, which directly impacts color and drip loss, and it may result in less tender pork chops (Saikia et al., 2024). Through nutritional manipulation is possible to modify the IMF levels. In swine production, various dietary strategies have been implemented to enhance IMF content. For example, it has been reported that dietary approaches aimed at increasing tissue fat saturation result in elevated IMF content and carcass fatness. Thus, lipid accumulation is positively associated to SFA concentration both in subcutaneous and IMF (Olivares et al., 2009). The dietary lipid sources consumed by pigs directly impact the fatty acid composition of pork (Nieto and Ros, 2012). In pigs, the fatty acid profile of muscle and adipose tissue are easily adjusted by altering the ratio of fatty acids in their diet, particularly by feeding diets abundant in PUFAs, which are mainly found in grains and oleaginous seeds (Wood et al., 2008). The focus on the nature of fat sources in pigs’ diets and the interest in modifying meat’s fatty acid composition derives from the fact that fatty acid composition is crucial in defining meat quality since it determines differences in sensory attributes and in the nutritional value for human consumption (Nieto and Ros, 2012). Dietary fats and oils give the diet a high energy value, and their fatty acid pattern is reflected in those of animal products (Alonso et al., 2012). The lipid content of bakery and confectionery products is higher compared to traditional feed ingredients and their fatty acid profile contains a significant amount of SFAs especially from butter and partially hydrogenated vegetable oils. Based on the previous considerations, it is appropriate to evaluate the effect on meat lipid composition, when balanced diets supplemented with FFPs are fed to animals producing meat (Gutiérrez-Luna et al., 2022). This study aimed to evaluate the effects of partially replacing 30% of conventional grains with the same amount of sugary or salty FFPs in swine diets. This effort is part of a larger study using FFPs in growing finishing pigs. Here, we report how high dietary FFPs inclusion affects the meat composition and technological and sensory characteristics of pork chops. Our hypothesis was that the lipid composition of FFPs may adversely impair pork quality in terms of fat firmness and meat flavor compared to the control diet.

## Material and Methods

The experimental plan was compliant with the Swiss regulation of animal welfare. The Swiss Federal Committee for Animal Care and Use of Canton Fribourg in Switzerland authorized the experiment (authorization code 2021-35-FR). The trial was carried out at the Agroscope Experimental Swine Research Centre in Posieux (Fribourg, Switzerland).

### Animals and Diets

The detailed rearing conditions, the performance traits, dietary treatments, and the slaughter conditions of the barrows used to collect the meat and adipose tissue samples were previously described by Mazzoleni et al. (2023). Briefly, 36 castrated Swiss Large White male piglets from five litters were involved. Starting from the grower period, pigs weighed 22.38 ± 1.70 kg (mean ± SD) and were assigned within litters to one of the three dietary groups: standard (**ST**), salty (**SA**), and sugary (**SU**). Pigs were reared in a single-group pen in which they could access to three automated feeders with a single space (Mastleistungsprüfung MLP-RAP; Schauer Agrotronic AG, Sursee, Switzerland). The animals were fed individually using an ear tag with identification chip which allowed each pig to receive the assigned diet from the assigned computerized feeder. The SA-FFPs diet was formulated with salty products such as crackers, pasta, bread, breadsticks, whereas the SU-FFPs diet included sugary products such as chocolate, breakfast cereals, and cookies. The FFPs were divided between salty and sugary considering the total sugar content, expressed in sucrose. The chemical composition of the pure SA and SU FFPs used to formulate the experimental diets are reported in Supplementary Table S1. The grower and finisher diets were formulated following the Swiss feeding recommendations for pigs (Agroscope, 2022; Table 1). The standard grower diet (ST-G) and the standard finisher diet (ST-F) were formulated considering a reference BW of 40 and 80 kg, respectively. For the SA and SU grower (SA-G and SU-G, respectively) and finisher (SA-F and SU-F, respectively) diets, a portion of conventional ingredients such as cereals and fats included in the ST-G and ST-F diets were replaced by 30% salty and sugary FFPs. Comprehensive details regarding the ingredients and their respective inclusion levels utilized in formulating the diets can be found in the study conducted by Mazzoleni et al (2023). The pigs had ad libitum access to fresh water and to the grower and finisher diets from 20 to 60 kg BW and from 60 kg BW to slaughter, respectively. The grower and finisher diets were formulated to be isoenergetic and isonitrogenous. All diets were prepared as pellets (<70 °C), and they included microbial phytase at 500 FTU/kg (0.16 digestible P/100 FTU). The fatty acid profiles of the dietary treatments differed (Table 1). The main difference in the saturated fatty acids (SFAs) was related to the 17:0 fatty acids, which were higher in the ST diet than in the SA and SU diets. Further, the monounsaturated fatty acid (MUFA) content of the diets differed, particularly regarding the 18:1n-9 content, for which the experimental diet had higher values than the ST diet. Finally, the polyunsaturated fatty acids (PUFA) content was higher in the ST diet than in the SA and SU diets (Table 1).

**Table 1.** Diet composition and nutrient and digestible energy content (g/kg or MJ/kg on DM) of the unsupplemented standard growing (ST-G) and finishing (ST-F) diets and the growing and finishing diets supplemented with 30% salty (SA-G and SA-F) or sugary (SU-G and SU-F) former food products fed to growing-finishing pigs.

Items	Dietary treatments^1^					
	Growing diets			Finishing diets		
	ST-G	SA-G	SU-G	ST-F	SA-F	SU-F
Crude fat	52	53	61	45	53	59
Crude protein	173	174	176	152	151	153
Crude fiber	42	40	39	42	39	40
Sodium	1.3	3.7	1.5	1.7	3.2	1.7
Total ash	68	74	72	62	65	64
Fatty acids profile, g/100 g total fatty acids						
SFA	34.1	21.9	34.0	34.4	19.9	31.9
12:0	0.00	0.16	1.25	0.00	0.00	1.17
14:0	1.21	0.36	1.54	1.13	0.30	1.56
15:0	0.26	0.00	0.11	0.24	0.00	0.18
16:0	22.8	17.3	21.8	23.5	16.1	21.0
17:0	0.53	0.00	0.25	0.51	0.00	0.00
18:0	8.93	3.05	8.11	8.65	2.74	7.21
20:0	0.22	0.29	0.33	0.23	0.27	0.30
22:0	0.16	0.45	0.25	0.17	0.40	0.24
24:0	0.00	0.22	0.11	0.00	0.19	0.15
MUFA	34.3	48.7	38.9	31.1	50.1	39.9
16:1	1.57	0.28	0.67	1.45	0.28	0.66
18:1 trans-11	2.28	0.92	1.28	2.11	0.89	1.23
18:1 cis-9	29.2	47.1	36.4	26.3	48.5	37.3
20:1 n-9	0.50	0.37	0.34	0.49	0.37	0.34
PUFA	31.6	29.4	27.1	34.5	30.0	28.2
18:2 n-6	28.3	27.1	24.9	31.6	28.0	26.0
18:3 n-3	2.94	2.35	2.33	2.79	2.05	2.16
MUFA/SFA ration	1.01	2.22	1.14	0.91	2.52	1.25
Calculated						
Digestible phosphorus, g/kg DM	2.9	2.9	2.9	2.2	2.2	2.2
Digestible lysine, g/kg DM	8.3	8.3	8.3	6.2	6.2	6.2
DE, MJ/kg DM	13.7	13.7	13.7	13.7	13.7	13.7

^1^All diets for the growing and finishing phases were formulated according to the energy and nutrient requirements of pigs with a BW of 40 and 80 kg, respectively (Agroscope, 2022).

Abbreviations: ST, standard diet; SA, salty former food diet; SU, sugary former food diet; DE, digestible energy.

### Slaughter procedure, sampling, and meat trait measurements

Pigs were slaughtered at the Agroscope research slaughterhouse after fasting for 16 h (Bee et al., 2017) when they reached ~110 kg BW. The animals were stunned with CO_2_, after which they were exsanguinated, scalded, mechanically dehaired, and eviscerated.

The pH and temperature of the longissimus dorsi (LD) were monitored at 45 min, 3 h, and 24 h *postmortem* using a Testo 205 pH meter (Testo, Mönchaltorf, Switzerland). Testo 205 is portable pH meters provided with pH and temperature probes and compensation is automatic. Moreover, the calibration was performed prior to each measurement series in conditions similar to those at which carcass was exposed. These measurements were performed at the 10th-rib level inside the intact left side of the carcass (Berard et al., 2008). At 24 h post-mortem, the LD from the left side of the carcass was cut between the 10th and 12th ribs to yield five 1.5 cm thick chops labelled from A to E. Subcutaneous fat was removed from chops.

Color and drip loss were measured at the end of the aging period on the chop A. After 20 min of blooming, L* (lightness), a* (redness), and b* (yellowness) values were measured three times in each muscle section using a CM-700d Chroma meter (Illumina D65, light source C, observer 10°, aperture 8 mm; Konica Minolta, Tokyo, Japan) in the CIE L*a*b* color space. Drip losses were measured as the proportions of purges generated during storage for 24 h at 2 °C (Honikel, 1998). Prior the drip losses measurements, the samples were suspended as described by Bee et al. (2007). Chops were vacuum packaged, kept for 2 d at 4 °C. Chops were leaved 3 days at 4 °C, dry blotted, weighted, and conditioned for drip loss (at the end of maturation) analysis. Forty-eight hours later, chops were dry blotted and weighted to determine purge loss. Then, bags were opened and chops were dry blotted, weighed, vacuum packaged, and stored at −20 °C until further analysis. Chops were allowed to thaw at 2 °C for a minimum of 24 h, then weighed to determine thaw loss on sections B and D. Subsequently, the LD chops were cooked on a 170 °C preheated Indu-griddle SH/GR 3500 grill plate (Hugentobler, Schönbül, Switzerland) to an internal temperature of 69 ± 2 °C measured by an internal temperature probe associated to the cooking plate. Cooking loss was calculated by reweighing the cooked samples. Ten cores of 1.27 cm diameter per sample were obtained parallel to the fiber orientation with an electrical drill. The cores were obtained and always sheared in the same starting from the medial end of the chop. Finally, cooked samples from 10 cores of the LD chops (5 per chop) with a diameter of 1.27 cm each were cooled to ambient temperature. Shear force was determined by using a Texture Analyzer TA. HDplus (Stable Micro Systems, Godalming, England) equipped with a 2.5-mm thick Warner Bratzler shear blade (4mm/sec). Shear force was measured perpendicularly to the fiber direction. The maximum shear forces of 10 cores per chop (two chops per animal) were recorded and averaged per animal. Sections C and E were used for sensory analysis as described below.

### Sample preparation for chemical analysis

#### Longissimus dorsi and backfat

All samples were freeze dried (Christ Delta 2-24, Kühner AG, Birsfelden, Switzerland) before analysis. After grinding with the Grindomix GM 200 (Retsch GmbH, Düsseldorf, Germany), dry matter (DM) content of LD samples was analysed by heating at 105˚C for 3 h. Ash was then determined by incineration at 550°C until reaching a stable mass according to ISO 5984_2002 (prepASH 229, Precisa Gravimetrics AG, Dietikon, Switzerland). Backfat samples were grinded with a mixer and dried at 105°C for 3 h for determining the DM content (ISO 5984:2002; prepASH 229, Precisa Gravimetrics AG, Dietikon, Switzerland).

### Chemical analysis

On feed samples, dry matter was determined gravimetrically after drying at 105 °C for 3 h. Ash content was determined after 3 h at 550 °C. The crude protein (CP) content (total N × 6.25) was analyzed with a LECO FP-2000 analyzer (Leco, Mönchengladbach, Germany; International Organization for Standardization; ISO, 2008). Feed samples were hydrolyzed in 10% (v/v) HCl for 1 h to determine the dietary crude fat content. The hydrolysate was dried and extracted with petroleum ether using the Büchi SpeedExtractor E 916 (Büchi Labortechnik AG, Flawil, Switzerland). The fatty acid profiles of the feed were determined in lyophilized samples as described by Ampuero Kragten et al. (2014). Briefly, lipids were transmethylated for 3 h at 70 °C using 5% methanolic HCl as an acid reagent. The methyl esters were neutralized with a potassium carbonate solution and purified on silica gel. Fatty acid methyl esters were analyzed by gas chromatography (6850 series; Agilent Technologies AG, Basle, Switzerland) equipped with a flame ionization detector (detector temperature 250 °C). Nonadecanoic acid methyl ester (19:0) was used as internal standard.

Crude fiber content was obtained gravimetrically (ISO 6865:2000) by incinerating residual ash after acid and alkaline digestions with a fiber analyzer (Fibretherm Gerhardt FT-12, C. Gerhardt GmbH & Co. KG, Königswinter, Germany).

Sodium content in the feed was analyzed according to EN 15510:2008 by ICP-OES (ICP-OES 5800, Agilent Technologies, Switzerland) after microwave digestion. Samples were dissolved in a glass tube (5 ml HNO_3_ 65% + 3 ml H_2_O ASTM Class I) using a microwave digester (UltraClave, MLS GmbH, Leutkirch, Germany) at 235 °C for 60 min (1,000 W). Before the analysis, the samples were diluted with HNO_3_ 2%.

On meat samples, protein content in the LD was analyzed using the Dumas method (ISO 16634-1:2008) by a LECO TruMac CNS-928 (Leco, Mönchengladbach, Germany), which was calculated as total N × 6.25. Fat content was determined with petrol ether after acid hydrolysis (ISO 6492:1999) and used to calculate the IMF content. Fatty acids in the LD and backfat were determined by transmethylation/esterification under acid catalysis (5% HCl in MeOH) at 70 °C for 3 h, as described by Ampuero Kragten et al. (2014). Briefly, depending on fat content, the samples were mixed with 0.25 to 2 ml of internal standard (C 19:0; nonadecanoic acid), 3 to 6 ml of HCl (5% in methanol), and between 0 and 1.75 ml of toluene. The reaction mix was neutralized using 6% K_2_CO_3_ and purified by solid-phase extraction. Fatty acids were determined using a gas chromatography instrument equipped with a flame ionization detector and a Supelcowax 10 polar column of 15 m × 0.1 mm, 0.1 μm of length (Agilent 6850, Agilent Technologies, Switzerland; Ampuero Kragten et al., 2014).

Amino acids in the LD and backfat were measured according to ISO 13903:2005. Briefly, after oxidation, 24-h acid hydrolysis was performed with 6 M HCl, followed by derivatization with AccQ-Tag Ultra reagent (Waters, Milford, MA, USA). Amino acid profile was determined by using ultra-high-performance liquid chromatography coupled with a UV detector (Vanquish Horizon, Thermo Scientific, Reinach, Switzerland).

### Meat sensory analyses

Two chops from the left LD were thawed at 4 °C for 24 h, sliced, and cooked for 10 min in a heating plate until the core temperature reached 69 ± 2 °C.

During cooking process pork, slices were rotated every minute and half to ensure even cooking. Then, once cooked, the slices were cut into 1 cm cubes. A total of 21 subjects (52% women; mean age: 26 ± 3 years) were recruited from employees and students of the Faculty of Agriculture and Food Sciences at the University of Milan (Italy). Only subjects who like, who regularly consume pork meat (at least once a week) and without food intolerances and allergies were selected. The study complied with the Declaration of Helsinki and was approved by the Ethics Committee of the University of Milan (protocol code: 92/22; date of approval: October 28, 2022). Signed informed consent was obtained from all the selected subjects. The participants attended nine training sessions at the Sensory and Consumer Science Laboratory (SCS Lab) of the Department of Food, Environmental and Nutritional Sciences of the University of Milan, designed according to ISO guidelines (ISO 8589, 2007).

A ‘‘difference from control’’ method was used (Meilgaard et al., 1999; Lawless and Heymann, 2010; Pagliarini, 2021). First, participants took part in six preliminary sessions to distinguish and define appropriate sensory attributes that characterize pork loins. After guided open discussions, redundant attributes were eliminated and the terms pork aroma, pork flavor, sweet taste, salty taste, and tenderness were selected during the sensory evaluation. During these sessions, the participants were instructed about the meaning of the sensory descriptors. Undoubtedly, sensory perception varies between individuals. However, the selection and training phases, as well as the participation at several preliminary sessions to learn how to use the scale and their respective extremes allowed to include in the final panel only judges able to provide robust results. Their performance was monitored during the experimental sessions. Subsequently, 18 pork loin samples (six loin chops from each dietary treatment selected based on similar chemical composition to reduce the intra-variability among samples for each treatment were evaluated in three different sessions (30 min/session).

Each subject was first presented with the control sample (loin from pig fed with ST diet). After tasting the ST sample, subjects had to evaluate the loin samples from SA and SU diet (doing a comparison with ST sample; Meilgaard et al., 1999). The presentation order of SA and SU samples was randomized by judges. Intensity of each sensory attribute was rated on a linear structured scale with the control samples as central value (score 0), whereas the extremes were ‘‘much less intense than the control” (left side of the scale; score −5) and “much more intense than the control” (right side of the scale; score + 5). Each sample was presented to the participants as two cubes of meat provided in plastic plates labelled with three-digit codes in a serving portion. The judges were instructed to remove the cover, smell, and taste the samples. Data acquisition was performed with Fizzv2.31 software (Biosystèmes, Couternon, France).

### Calculations and statistical analysis

Statistical analyses of meat quality traits and meat chemical composition were conducted using R software (Version 4.2.1). The results were analyzed by ANOVA, and the model contained the dietary treatment (ST, SU, and SA) as a fixed effect and the litter of origin as a random effect. Only for the data about to LD color parameters over time was a linear mixed-effects regression (Lme4) model used, including the dietary treatment and the time (24 h vs. 72 h), and the two-way interaction was considered fixed effects and the animal as a random effect. For pairwise comparisons, the Sidak function was performed using a modified Tukey test for multiple comparisons of means. Means and pooled SEM were calculated with the *lsmeans* function from the *emmeans* package (Lenth, 2016). The residuals of the linear mixed-effects models were checked for normality and homoscedasticity. Variables that did not follow a normal distribution (fatty acids in IMF and backfat) were subjected to logarithmic transformation before the data analysis. Values are presented as ls-means with their standard errors. A *P*-value < 0.05 was considered significant while a *P*-value < 0.10 was considered a tendency.

Sensory data were subjected to analysis of variance (ANOVA) considering treatments, judges, sessions as fixed factors and sensory attributes ratings as dependent variables. The interaction judges*sessions has been also evaluated to check judges’ performance across sessions. Differences among samples according to dietary treatment (SU vs. ST; SA vs. ST) were evaluated through Dunnett test. A *P*-value of <0.05 was considered significant. The statistical analysis was carried out using XLSTAT (Version 2019.2.2, Addinsoft, Boston, MA, USA).

## Results

The fatty acid profiles of the dietary treatments differed (Table 1). The main difference in the saturated fatty acids (SFAs) was related to the 17:0 fatty acids, which were higher in the ST diet than in the SA and SU diets. Further, the monounsaturated fatty acid (MUFA) content of the diets differed, particularly regarding the 18:1n-9 content, for which the experimental diet had higher values than the ST diet. Finally, the polyunsaturated fatty acids (PUFA) content was higher in the ST diet than in the SA and SU diets (Table 1).

### Protein content and amino acid composition of the longissimus thoracis

The protein content and amino acid composition of the LD in pigs fed the ST, SA, or SU diets are summarized in Table 2. Except for the tendency of a lower (*P* = 0.08) cysteine content in the SA pigs compared to ST and SU pigs, the dietary treatment had no effect (*P *> 0.05) on the protein, the essential (EAA) and non-essential (NEAA) amino acid content of the LD. Concordantly, the ratios of EAA to NEEA, as well as the levels of flavor-enhancing amino acids, were similar in the three treatment groups (Table 2).

**Table 2.** The amino acid composition of the longissimus thoracis muscle from pigs fed either a basal grower-finisher diet or the basal diet with 30% salty (SA) or sugary (SU) former food products.

Items	Dietary treatments^1^				
	ST	SA	SU	SEM	*P*-values
Total protein	82.60	80.00	81.10	0.671	0.291
*Essential amino acids (EAA)*					
Arginine (Arg)	5.00	4.89	4.95	0.041	0.292
Histidine (His)	3.30	3.20	3.20	0.032	0.271
Isoleucine (Ile)	3.90	3.80	3.80	0.031	0.314
Leucine (Leu)	6.30	6.20	6.30	0.052	0.245
Lysine (Lys)	6.90	6.70	6.80	0.061	0.257
Methionine (Met)	2.20	2.10	2.10	0.021	0.236
Phenylalanine (Phe)	3.10	3.00	3.10	0.032	0.291
Threonine (Thr)	3.45	3.39	3.40	0.033	0.263
Valine (Val)	4.13	3.99	4.03	0.042	0.262
EAA^2^	38.80	37.50	37.90	0.301	0.264
*Non-essential amino acids (NEAA)*					
Alanine (Ala)	4.40	4.20	4.30	0.041	0.269
Asparagic acid (Asp)	7.39	7.14	7.30	0.072	0.293
Cysteine (Cys)	1.00	0.98	1.00	0.011	0.082
Glutamic acid (Glu)	12.20	11.80	11.90	0.112	0.344
Glycine (Gly)	3.40	3.30	3.30	0.032	0.244
Proline (Pro)	2.87	2.80	2.83	0.021	0.321
Serine (Ser)	2.88	2.78	2.81	0.020	0.212
Tyrosine (Tyr)	2.88	2.77	2.82	0.030	0.221
NEAA^3^	37.00	35.80	36.30	0.320	0.270
Total amino acids	75.80	73.30	74.20	0.601	0.267
EAA/NEAA	1.05	1.05	1.04	0.101	0.536
Flavour amino acids^4^	32.50	31.40	31.70	0.281	0.295

^1^All diets for the growing and finishing phases were formulated according to the energy and nutrient requirements of pigs with a BW of 40 and 80 kg, respectively (Agroscope, 2022).

^2^EAA = Lys + Met + Thr + Val + Leu + Ile + Tyr + Phe + His + Arg.

^3^NEAA = Arg + His + Asp + Glu + Ala + Pro + Ser + Cys.

^4^Flavor amino acids = Glu + Asp + Ala + Arg + Gly.

Data are expressed as % of the dry meat weight.

Abbreviations: ST, standard diet; SA, salty former food diet; SU, sugary former food diet.

### Meat quality, meat chemical composition, and fatty acids profile of the intramuscular fat

The three dietary treatments had no effect on meat quality traits (Table 3). Similarly, the dietary treatments did not affect the color parameters measured in the LD samples at 24 and 72 h post-mortem under vacuum refrigerated storage (Table 4), whereas storage time did (*P* < 0.05). The meat lightness (L*), redness (a*), yellowness (b*), and saturation (C*) increased (*P *≤ 0.01) after 72 h, while hue angle (H*) decreased (*P *< 0.05).

**Table 3.** Meat quality traits from pigs fed either a basal grower-finisher diet or the basal diet with 30% salty (SA) or sugary (SU) former food products

Items	Dietary treatments^1^				
	ST	SA	SU	SEM	*P*-values
pH_45min_	6.70	6.80	6.70	0.051	0.391
pH_3h_	6.50	6.60	6.50	0.072	0.583
pH_24h_	5.40	5.40	5.40	0.021	0.472
*T*_45min_, °C	34.30	34.70	33.60	0.780	0.566
*T*_3h_, °C	20.80	21.00	20.70	0.342	0.834
*T*_24 h_, °C	5.00	4.90	4.70	0.171	0.545
Thawing loss, %	6.00	5.40	5.70	0.311	0.413
Cooking loss, %	21.50	21.30	21.10	0.732	0.952
Drip, %	2.30	2.30	2.80	0.263	0.271
WBSF, N	51.10	47.70	48.90	2.351	0.583

^1^All diets for the growing and finishing phases were formulated according to the energy and nutrient requirements of pigs with a BW of 40 and 80 kg, respectively (Agroscope, 2022).

Abbreviations: ST, standard diet; SA, salty former food diet; SU, sugary former food diet.

**Table 4.** Effect of a basal grower-finisher diet or the basal diet with 30% salty (SA) or sugary (SU) former food products and storage time on porcine longissimus thoracis color

Time	Dietary treatments^1^							*P*-values^2^		
	ST		SA		SU					
	24 h	72 h	24 h	72 h	24 h	72 h	SEM	*D*	*T*	*D* × *T*
L*	56.1	58.5	56.3	59.8	56.5	59.4	0.63	0.775	<0.001	0.297
a*	0.8	1.2	0.9	1.2	0.8	1.1	0.09	0.745	0.001	0.838
b*	11.9	13.2	11.8	13.4	11.7	13.1	0.21	0.571	<0.001	0.561
C*	12.3	13.7	12.1	13.8	11.9	13.5	0.24	0.611	<0.001	0.681
H*	77.9	75.6	77.6	76.3	78.5	76.6	0.01	0.663	0.021	0.547

^1^All diets for the growing and finishing phases were formulated according to the energy and nutrient requirements of pigs with a BW of 40 and 80 kg, respectively (Agroscope, 2022).

^2^ *P*-values for the effect of the dietary treatment (*D*), time of storage (*T*), and of the *D* × *T* interaction.

Abbreviations: ST, standard diet; SA, salty former food diet; SU, sugary former food diet.

Accordingly, both the SA and SU diets affected the fatty acid composition of intramuscular fat (Table 5). Regarding SFAs, only 17:0 was affected by the dietary treatment, with higher values in the ST (*P* < 0.01) than in the SA and SU groups. Total MUFA content was higher in the SA (*P* < 0.01) than in the ST and SU groups. This difference was mainly due to the higher content of 18:1n-9 fatty acid in the SA (*P* < 0.01) than the ST and SU groups, while both 17:1cis-10 and 18:1trans-11 levels were lower (*P* < 0.01) in the SA compared to the ST group with the SU group being intermediate. The content of PUFA in the IMF of the pigs fed the SA diet tended to be lower (*P *= 0.08) than in the ST pigs. In particular, the 18:3n-3, 20:5n-3, and 22:5n-3 levels were lower in the IMF of pigs fed the SA diet (*P* < 0.01) than those fed the ST diet. Compared to the ST, the SU showed a lower level of 18:3n-3 fatty acid (*P* < 0.01). The SA diet caused an increased MUFA/SFA ratio (*P *= 0.02) and a decreased sum of n-3 fatty acids (*P* < 0.01) compared to the ST and SU diets. Thus, the SA diet led to the highest value of n-6/n-3 ratio, while the lowest value was found in the IMF of pigs fed the ST diet.

**Table 5.** Fatty acid profile (g/100 g total fatty acids) in the intramuscular fat from pigs fed either a basal grower-finisher diet or the basal diet with 30% salty (SA) or sugary (SU) former food products

Items	Dietary treatments^1^				
	ST	SA	SU	SEM	*P*-values
Intramuscular fat, g/kg muscle	40.10	47.90	44.10	6.611	0.351
Fatty acid profile, g/100 g total fatty acids					
SFA	38.30	37.50	38.50	0.221	0.209
10:0	0.08	0.09	0.07	0.005	0.514
12:0	0.10	0.10	0.10	0.002	0.178
14:0	1.29	1.25	1.31	0.016	0.390
16:0	24.30	24.10	24.50	0.125	0.575
17:0	0.17^b^	0.14^a^	0.15^a^	0.003	<0.001
18:0	12.10	11.70	12.10	0.133	0.306
MUFA	53.00^a^	54.80^b^	53.40^a^	0.228	0.002
16:1n-7	3.38	3.10	3.21	0.075	0.320
16:1cis-3	0.30	0.32	0.30	0.008	0.584
17:1cis-10	0.24^b^	0.18^a^	0.19^a^	0.006	<0.001
18:1trans-11	4.25^b^	3.86^a^	3.99^ab^	0.054	0.008
18:1n-9	43.70^a^	46.50^b^	44.60^a^	0.251	<0.001
PUFA	8.75	7.65	8.11	0.203	0.081
18:2n-6	5.99	5.38	5.67	0.127	0.148
18:3n-6	0.06	0.05	0.04	0.003	0.067
20:3n-6	0.16	0.14	0.15	0.005	0.099
20:4n-6	1.12	0.95	1.02	0.047	0.353
20:2n-6	0.19	0.19	0.19	0.003	0.886
22:4n-6	0.15	0.14	0.14	0.004	0.380
18:3n-3	0.38^b^	0.30^a^	0.34^a^	0.008	<0.001
20:3n-3	0.07	0.05	0.06	0.002	0.056
20:5n-3	0.08^b^	0.05^a^	0.06^ab^	0.003	<0.001
22:5n-3	0.17^b^	0.11^a^	0.14^ab^	0.007	<0.001
MUFA/SFA ratio	1.39^a^	1.46^b^	1.39^a^	0.013	0.022
PUFA/SFA ratio	0.23	0.20	0.21	0.006	0.196
Sum of n-3 fatty acids^3^	0.71^b^	0.52^a^	0.60^b^	0.017	<0.001
Sum of n-6 fatty acids^2^	7.68	6.84	7.22	0.184	0.180
n-6/n-3 fatty acid ratio	10.80^a^	13.10^c^	12.00^b^	0.201	<0.001

^1^All diets for the growing and finishing phases were formulated according to the energy and nutrient requirements of pigs with a BW of 40 and 80 kg, respectively (Agroscope, 2022).

^2^n-3 fatty acids = 18:3 (cis-9,12,15-octadecatrienoic acid), 20:3 (cis-11,14,17-eicosatrienoic acid), 20:5 (cis-5,8,11,14,17-eicosapentaenoic acid), 22:5 (cis-7,10,13,16,19-docosapentaenoic acid).

^3^n-6 fatty acids = 18:2 (cis-9,12-octadecadienoic acid), 18:3 (cis-6,9,12-octadecatrienoic acid), 20:2 (cis-11,14-eicosadienoic acid), 20:3 (cis-8,11,14-eicosatrienoic acid), 20:4 (cis-5,8,11,14-Eicosadienoic acid), 22:4 (cis-7,10,13,16-docosatetraenoic acid).

Abbreviations: ST, standard diet; SA, salty former food diet; SU, sugary former food diet.

### Fatty acid composition of the backfat

The SA diets significantly decreased the total SFAs content in the backfat (*P* < 0.01) compared to both the ST and SU diets (Table 6). In particular, the content of 14:0, 15:0, 16:0, 17:0, and 18:0 was lower in the SA than in the ST group (*P* < 0.01), whereas the SU diet increased the abundance of the 12:0, 14:0, and 15:0 (*P* < 0.01) compared to the SA diet. The MUFA content also differed between the three experimental diets (*P* < 0.01), with the lowest value in the ST and the highest in the SA group.

**Table 6.** Fatty acid profile (g/100 g total fatty acids) in the carcasses’ backfat from pigs fed either a basal grower-finisher diet or the basal diet with 30% salty (SA) or sugary (SU) former food products

Item	Dietary treatments^1^				
	ST	SA	SU	SEM	*P*-values
SFA	41.3^b^	38.1^a^	40.0^b^	0.33	<0.001
10:0	0.05	0.04	0.05	0.002	0.646
12:0	0.07^a^	0.07^a^	0.13^b^	0.005	<0.001
14:0	1.21^b^	1.07^a^	1.31^c^	0.021	<0.001
15:0	0.06^b^	0.04^a^	0.06^b^	0.002	0.001
16:0	24.2^b^	22.8^a^	23.4^a^	0.15	<0.001
17:0	0.35^b^	0.26^a^	0.31^ab^	0.102	0.001
18:0	15.1^b^	13.6^a^	14.5^ab^	0.23	0.015
20:0	0.23	0.22	0.23	0.004	0.707
MUFA	47.4^a^	50.8^c^	48.8^b^	0.31	<0.001
14:1n-5	0.02^b^	0.01^a^	0.02^b^	0.001	<0.001
16:1n-7	1.85^b^	1.39^a^	1.53^a^	0.051	<0.001
17:1n-10	0.35^b^	0.23^a^	0.28^a^	0.013	<0.001
t18:1n-7	2.75^c^	2.02^a^	2.24^b^	0.059	<0.001
18:1n-9	40.9^a^	45.8^c^	43.2^b^	0.38	<0.001
19:1n-9	0.08^b^	0.05^a^	0.07^ab^	0.004	0.034
20:1n-9	1.09	1.16	1.21	0.026	0.172
PUFA	11.2	11.0	11.1	0.10	0.830
18:2n-6	8.59	8.85	8.72	0.079	0.422
18:3n-6	0.02	0.01	0.01	0.001	0.495
20:2n-6	0.45	0.45	0.45	0.007	0.998
20:3n-6	0.062^b^	0.054^a^	0.059^ab^	0.0011	0.005
20:4n-6	0.16	0.16	0.15	0.003	0.117
22:4n-6	0.05	0.05	0.05	0.002	0.878
18:3n-3	0.76^c^	0.62^a^	0.69^b^	0.013	<0.001
20:3n-3	0.14^b^	0.11^a^	0.13^b^	0.003	<0.001
20:5n-3	0.01	0.01	0.01	0.002	0.462
22:5n-3	0.06	0.04	0.05	0.002	0.074
PUFA/SFA ratio	0.27	0.29	0.28	0.004	0.171
MUFA/SFA ratio	1.15^a^	1.33^b^	1.22^a^	0.017	<0.001
Sum of *n-3* fatty acids^2^	0.96^c^	0.78^a^	0.88^b^	0.017	<0.001
Sum of *n-6* fatty acids^3^	9.34	9.59	9.45	0.083	0.477
n-6/n-3 fatty acid ratio	9.72^a^	12.30^c^	10.73^b^	0.21	<0.001

^1^All diets for the growing and finishing phases were formulated according to the energy and nutrient requirements of pigs with a BW of 40 and 80 kg, respectively (Agroscope, 2022).

^2^n-3 fatty acids = 18:3 (cis-9,12,15-Octadecatrienoic acid), 20:3 (cis-11,14,17-Eicosatrienoic acid), 20:5 (5Z,8Z,11Z,14Z,17Z-eicosa- 5,8,11,14,17-pentenoic acid), 22:5 (cis-7,10,13,16,19-docosapentaenoic acid).

^3^n-6 fatty acids = 18:2 (cis-9,12-octadecadienoic acid), 18:3 (cis,cis,cis-6,9,12-octadecatrienoic acid), 20:3 (cis-8,11,14-eicosatrienoic acid), 20:2 (cis-11,14-eicosadienoic acid), 20:4 (cis-5,8,11,14-eicosadienoic acid), 22:4 (cis-7,10,13,16-docosatetraenoic acid).

Abbreviations: ST, standard diet; SA, salty former food diet; SU, sugary former food diet.

With the exception of 20:1n-9, all MUFA analyzed were significantly influenced by diet, as shown in Table 6. The highest total MUFA value was found in the SA group, followed by SU and ST. However, this difference was mainly due to the 18:1n-9 fatty acid, which was higher in the SA group than in the ST and SU groups. By contrast, all other MUFA followed an opposite trend, with a lower abundance in the SA group than in the ST group. The SU backfat also differed from the SA in the MUFA profile, specifically for its higher levels of 14:1n-5 and t18:1n-7 (Table 6).

Similar to the PUFA profile of the IMF, 18:3n-3, 20:3n-6, and 20:3n-3 were less abundant (*P* < 0.01) in the SA compared to the ST and SU groups. The SA diet also increased (*P* < 0.01) the MUFA/SFA ratio compared to the other dietary treatments. The sum of the n-3 fatty acids and the n-6/n-3 fatty acids ratio were affected (*P* < 0.01) by both the SA and SU diets. In particular, the SA group had the lowest levels of n-3 fatty acids, and the ST group had the highest levels. Consequently, the SA group showed the highest value of n-6/n-3 ratio while the ST diet had the lowest value.

### Meat sensory attributes: salty and sugary former food vs. standard diets

**Figure 1.**
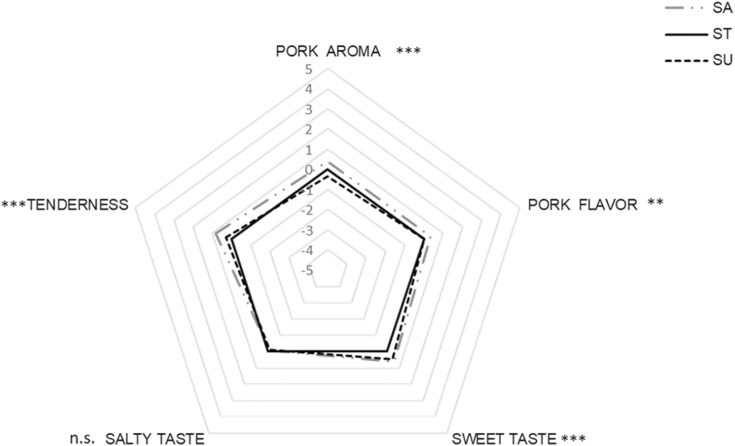
Mean values of sensory attributes for samples derived from pigs fed the sugary (SU) and salty (SA) diets compared to the samples derived from pigs fed standard diets (ST). n.s. not significant; **P < 0.01; ***P < 0.001.

ANOVA results depicted that the interaction ‘judges * sessions’ was not significant for any of attributes considered (pork aroma: F = 0.89, *P* = 0.67; pork flavor: F = 1.02, *P* = 0.43; sweet taste: F = 0.93, *P* = 0.60; salty taste: F = 0.87, *P* = 0.69; tenderness: F = 0.76, *P* = 0.85), confirming judges’ reliability throughout sessions. A significant treatment effect (Fig. 1) was found for pork aroma (F = 12.83, *P* < 0.001), pork flavor (F = 4.54, *P* < 0.01), sweetness (F = 15.33, *P* < 0.001), and tenderness perception (F = 14.31, *P* < 0.001). No differences according to dietary treatment has been highlighted for salty taste (F = 0.87, *P* = 0.69).

As reported in Table 7, the SA sample exhibited a significant greater intensity in terms of pork aroma and pork flavor compared to the ST sample. Sample obtained from pork feed by sugary formed food (SU) was perceived as significantly less intense than ST in term of pork aroma. Moreover, both SU and SA samples were perceived as sweeter compared to ST sample. As regard texture perception, SA sample was described as more tender than the ST sample.

**Table 7.** Mean values of sensory attributes of pork from pigs fed basal diet with 30% salty (SA) or sugary (SU) former food products compared to the reference pork from pigs fed basal grower-finisher diet

Sensory attributes	Treatment	*P*	Treatment	*P*
	ST vs SA		ST vs SU	
Pork aroma	0.37	0.012	−0.34	0.031
Pork flavor	0.36	0.011	0.00	1
Sweet	0.60	<0.001	−0.46	<0.001
Salty	0.11	0.620	0.07	0.790
Tenderness	0.84	<0.001	−0.30	0.110

Abbreviations: ST, standard diet; SA, salty former food diet; SU, sugary former food diet.

## Discussion

### Meat quality traits and fatty acid composition

In this study, the replacement of 30% of common energy sources by salty or sugary FFPs in the grower and finisher periods did not affect pig meat quality traits, such as pH, temperature, water holding capacity, shear force, and color. To our knowledge, this is the first study to evaluate the effects of FFPs on meat quality in pigs. Similar studies have been performed in pigs fed food waste products different from FFPs because of their nature, processing requirements, safety, and legislation status (Pinotti et al., 2021). For example, Kjos et al. (2000) investigated the effects of waste products such as food leftovers, food-processing plants and bakery waste, and dairy waste in diets for growing-finishing pigs on growth performance, carcass characteristics, and meat quality. The authors observed that increasing the levels of food waste products from 20% to 100% of the dietary net energy content reduced the fat firmness and lightness (L* values) of both backfat and loin. Further, in Kjos et al.’s (2000) study, the proportion of SFAs decreased, while the PUFA level increased, but the sensory quality of the loin muscle was not affected. Biondi et al. (2020) observed that feeding pigs with tomato processing waste reduced the intramuscular fat, SFAs, and MUFA content and increased the n-6/n-3 fatty acid ratio in the intramuscular fat of pork. Kwak and Kang (2006) tested the effect of including 25 or 50% food waste and bakery by-products mixture (FWM) into a pig diet. They found that the experimental diets did not affect carcass characteristics (carcass weight, dressing percentage, backfat thickness and carcass grade), meat fatty acid composition, meat quality (marbling score, pH, water holding capacity, drip loss, L*, a*, b* values, Warner-Bratzler shear force, cooking loss), and taste panel test (flavor, taste, tenderness, juiciness,–and overall acceptance) compared with feeding a corn-soy diet. However, meat color was judged by the panel test as paler for 50% FWM fed animals than a corn-soy diet fed animals. Meat color was the only limiting factor when FWM was fed to finishing pigs (Kwak and Kang, 2006).

Our study showed different results for meat quality traits with FFPs compared to food waste. In fact, including up to 30% FFPs had no detrimental effects on the color and lightness of the loin muscle. However, there might be interest in increasing the FFPs inclusion level from the perspective of further reducing the use of grains and consequently the feed-food competition. In the present study, we chose an inclusion of 30% of FFPs in pig’s diet because, as reported in studies testing the effects of the bakery meal on animals, a level of FFPs inclusion higher than 30% could lead to detrimental effects on growth performance in pigs, as observed by Luciano et al. (2022) in weaned pigs. To our knowledge, there are no data about the effects of inclusion levels of FFPs higher than 30% in growing-finishing diets.

In the present study, the inclusion of 30% FFPs did not affect the accumulation of IMF in meat. Despite controversial opinions, there is literature reporting that the presence of certain levels of IMF contributes to a proper juiciness, tenderness, and flavor to the meat, and it is therefore desirable for the consumer’s acceptability (Lawrie and Ledward, 2014). The Swiss Large White breed is known to have an IMF content of about 3%, which is considered optimal from a taste point of view in Europe (Font-i-Furnols et al., 2012). In the present study IMF reached about 4% in all experimental groups, which is in line with values normally observed in pork of pigs reared in our experimental station (Ewaoluwagbemiga et al., 2023). Although IMF content was similar among diet groups in this study, dietary treatment did affect the IMF and backfat fatty acid profile, with smaller effects on the IMF than on the backfat. For instance, the relative content of SFA was unaffected in the IMF but decreased in the backfat of pigs fed the SA diet compared to pigs fed the ST and SU diets. It is known that the fatty acid profile of pork fat generally mirrors that of the diet (Wood et al., 2008). However, the majority of SFA is derived from de novo synthesis (Nakamura and Nara, 2004); thus, the lower SFA content in the backfat of the SA group cannot be completely explained by the correspondingly lower SFA content of the SA diet in both the growing and finishing phases compared to the SU and ST diets.

Stearoyl CoA desaturases (SCD), also known as delta-9 desaturase, are an essential component of de novo lipogenesis, as they catalyze the conversion of SFA to MUFA, which are key substrates for the formation of complex lipids, such as triglycerides and cholesterol esters (Flowers and Ntambi, 2009). The mRNA expression and activity of desaturase and elongase enzymes are influenced by numerous dietary components, including macronutrients (dietary fat, carbohydrates, and proteins), micronutrients (folate, vitamin B-12, and vitamin A), and polyphenols (resveratrol and isoflavones) (Gonzalez-Soto and Mutch, 2021). In particular, dietary PUFAs such as 18:2n–6 have been observed to decrease liver SCD activity, but SFA and MUFA do not (Gonzalez-Soto and Mutch, 2021). In our study, both the SA and SU diets resulted in a lower PUFA content, especially 18:2 n-6, than the ST diet. The low dietary PUFA content of the SU and SA diets did not suppress SCD activity, which desaturates SFA. This explains the lower SFA content and higher MUFA content in the backfat of SA pigs compared to ST pigs. However, the backfat of the SU pigs only had a higher MUFA content, while the amount of SFA was only numerically and not statistically lower than that of the ST pigs. We speculate that this discrepancy may be due to inter-animal variability, but this aspect merits further investigation.

This effect was observed only in backfat and not in IMF, probably because IMF is generally more unsaturated due to the greater amount of phospholipids (Yi et al., 2023). Compared to the ST and SU groups, the SA diet increased the relative MUFA content in both IMF and backfat. In the IMF, this effect was due to the increased abundance of oleic acid (18:1n-9). This result is consistent with the higher content of 18:1n-9 in the SA diet compared to the other experimental diets. These results corroborate those of Martins et al. (2018) and Navarro et al. (2021), who showed that supplementing pig diets with oleic acid-rich oils increased the 18:1n-9 in pork fat. A higher oleic acid content and a high MUFA/PUFA ratio in the IMF have been associated with an improved release of pleasant aromatic notes from Maillard reactions in cooked pork due to the lipid-Maillard interaction (Navarro et al., 2021). The latter could explain, at least in part, the results observed in the sensory analysis, in which the SA diet improved the sensory attributes of the LD compared to the ST. Similarly, the SU diet also resulted in a higher MUFA content in the backfat compared to the ST diet. Further, the meat from the SU pigs was perceived as sweeter compared to those from ST pigs. The effects of the SA diet on omega-3 fatty acids resulted in a lower n-3 content and a higher n-6/n-3 fatty acid ratio in both IMF and backfat. Compared to the ST group, the SU group followed the same trend as the SA group.

The 18:3n-3 fatty acid, also known as alpha-linolenic acid, is an essential fatty acid and must be obtained in the diet. 18:3n-3 is also a precursor of the longer chain n-3 PUFA, 20:5n-3, and the 22:5n-3 fatty acids, also known as eicosapentaenoic acid and docosapentaenoic acid, respectively (Sinclair et al., 2002). Accordingly, the lower content of 18:3n-3 in the SA diet compared to the ST diet (Table 4) led to a reduced amount of the same fatty acid in the IMF and, consequently, lower levels of the derived fatty acids (20:5n-3 and 22:5n-3). Similarly, the lower amount of 18:3n-3 in the backfat of pigs fed the SA diet is probably due to its reduced intake from the diet. This also explains the lower abundance of the 20:3n-3 fatty acid in the backfat of SA pigs, as this fatty acid is known to be a “dead-end” elongation product of 18:3n-3 (Berger and German, 1990). The 20:3n-6 fatty acid (dihomo-gamma-linolenic acid) is a PUFA normally present in mammals at low levels, and its initial precursor is the 18:2n-6 fatty acid (Mustonen and Nieminen, 2023). Again, the lower amount of the 20:3n-6 fatty acid in the SA pigs compared to the ST pigs is probably due to the lower content of the 18:2n-6 fatty acid in the SA diets. Taken together, these differences also explain the lower n-3 content and, consequently, the higher n-6/n-3 ratio in IMF and backfat of the SA-FFP-fed pigs compared to the ST. Although the high content of n-3 fatty acids is desirable in pork because of its potential beneficial effects on human health, increasing the n-3 content in pork could be problematic due to the off-odors and flavors resulting from the oxidation of the PUFA and consequently represent a challenge in food processing and storage (Wood et al., 2004).

Our results also showed that both 18:3n-3 and 18:2n-6 PUFA introduced by the diet were higher in the backfat than in the IMF, independent of the diet. This is in line with previous studies (Bee et al., 2002; Nguyen et al., 2003) and could be explained by the differences in the degree of incorporation of these PUFA into tissues. In particular, the intake of both 18:3n-3 and 18:2n-6 PUFA is probably higher than required, and part of these PUFA are stored in adipose tissue. The n-6/n-3 and PUFA/SFA ratios seem to play an important role, with several evidence indicating that diets with high n-3 PUFA content and low n-6/n-3 PUFA ratio could be more beneficial to human health (Dugan et al., 2015; O’ Connell et al., 2017; Lee et al., 2018; Minelli et al., 2023). Specifically, values of n-6/n-3 ratio ranging from 1:1 to 5:1 positively affect lipid metabolism and inflammation, and are considered protective against degenerative pathologies; however, modern Western diets typically have values from 15:1 to 20:1 (Duan et al., 2014). FFPs are produced starting with ultra-processed foods commonly used in Western diets. Accordingly, both the SU and the SA experimental diets increased the n-6/n-3 ratio in both loin muscle and backfat compared to the ST diet, with the SA diet providing the highest value of the ratio. The PUFA content in pork is only dependent on the dietary PUFA content (Bee et al., 2002), therefore the higher n-6/n-3 ratios observed in the SA and SU groups can be attributed to the FFPs. It is known that the higher the dietary n-6/n-3 ratio, the higher the metabolic health risk (Hibbeln et al., 2006). This effect of FFP-based diets on pork should still be investigated in detail, although pork has a high n-6/n-3 PUFA ratio even when animals are fed typical feed ingredients (Nong et al., 2020).

However, the meat and the backfat of the SA-fed pigs had a higher MUFA/SFA ratio, compared to the pigs of the ST and SU dietary groups. The higher MUFA/SFA ratio in the SA diet reflects the finisher diets’ composition, with higher SFA and lower MUFA content in the ST and SU finisher diets, compared to the SA. Based on previous findings and considering that the SA and SU diets have 3% and 1% higher levels of n-6 than the control diet, the differences in IMF and backfat observed between the dietary groups concerning the MUFA/SFA and n-6/n-3 ratio were not negligible. However, it could be speculated that the level of IMF is too low for this ratio to be harmful to human health.

### FFPs and their impacts on sensory attributes of pork

Substituting common energy sources with sugary FFPs in the SU diet resulted in pork with more perceived sweetness than pork from the ST-fed pigs. Similarly, including salty FFPs in pig diets generated a dual effect on the sensory attributes of pork, leading to increased tenderness and sweetness compared to pork from the ST-fed pigs. Moreover, salty FFPs in pig diets led to an increase in both pork aroma and flavor. The fatty acid composition of the LD muscle has been suggested to influence the eating quality of pork. The sweetness of the meat is generally influenced by meat marbling, which consumers perceive as sweeter due to enhanced flavor and liking (Ngapo et al., 2012). Moreover, free amino acids such as Gly, Ala, Ser, Thr, Pro, and Hyp and higher amounts of oleic acid-derived compounds are known to be associated with the sweet flavor of pork (Hoa et al., 2021; López-Martínez et al., 2023). In the present work, the dietary treatments did not affect the level of IMF accumulation and the abundance of sweet flavor-related amino acids in the loins. However, the content of oleic acid was significantly higher in the IMF of SA group compared to ST group. Although not significant, oleic acid content was numerically higher also in the SU group when compared to ST, which may have contributed to the sweeter perception. The IMF in pork has also been found to positively influence meat juiciness and tenderness (Junior et al., 2024). However, some studies did not find such a relationship, and the correlation between IMF and the sensory quality of pork remains controversial (Ngapo et al., 2012). As reported in the literature, the main determinants of meat tenderness beyond the IMF content are connective tissue and the proteolysis of key muscle proteins, which minimizes the loss of water-holding capacity determining its tenderness (Van Laack et al., 2001). No difference in shear force values was observed, therefore the effect of SA and SU diets on the proteolysis kinetics of the myofibrillar structure was not considered in this study.

Despite the different fatty acid profile of the diets, meat quality and intramuscular fat accumulation were similar between the three experimental groups. This lack of difference in meat quality parameters despite different fatty acid compositions aligns with previous studies, in which two diets differing in fatty acid composition did not lead to significant differences in overall meat quality traits and flavor precursors, despite differences in lipid composition and sensory attributes (Tikk et al., 2007). Similarly, previous studies (Bee et al., 2008; Tretola et al., 2019b) have showed that feeding growing-finishing pigs with diets with different supplementation levels of fatty acids did not influence the general meat qualitative traits.

According to our study, the IMF of the SA group showed a higher MUFA/SFA ratio than that of the ST group. In this regard, we speculated that the SFA content, particularly 18:0, 18:2, and the MUFA/SFA ratio could affect fat consistency (Ospina-E et al., 2012). Contrary to unsaturated FAs, SFA strongly influences the solid fat content of lipids, which expresses the solid fraction amount of lipids at each temperature, because of their high melting points (Hugo and Roodt, 2007). The literature reports that the higher content of several saturated fatty acids (SFA) in the backfat of ST pigs compared to SA pigs is positively correlated with fat firmness (Wood et al., 2008). By including 30% SU and SA FFPs in pig diets, no negative impact on meat quality characteristics was observed. By contrast, the inclusion of FFPs altered the fatty acid profile of the meat and backfat, resulting in an increased n-6/n-3 fatty acid ratio.

The sweetness of the meat was found to increase with both the SU and SA-FFPs. However, the tenderness of the meat was only higher when pigs were fed SA-FFPs. Additionally, the perception of pork aroma and flavor was also more intense with the SA-FFPs.

The lack of detrimental effects on meat quality traits is a positive outcome for including 30% FFP levels in diets of growing finishing pigs.

## Supplementary Material

skae070_suppl_Supplementary_Table_1

## Abbreviations

*a**: redness
*b**: yellowness
C*: saturation
CP: crude protein
EAA: essential amino acids
FFP: former foodstuff products
H*: hue angle
IMF: intramuscular fat
*L**: lightness
LD: *longissimus dorsi*
MUFA: monounsaturated fatty acid
NEAA: non-essential amino acids
PUFA: polyunsaturated fatty acids
SCD: stearoyl CoA desaturases
SFA: saturated fatty acid
WBSF: Warner–Bratzler shear force

## References

[CIT0001] Agroscope. 2022. Fütterungsempfehlungen und Nährwerttabellen für Schweine (Feeding recommendations and nutrient tables for pigs). Posieux, Switzerland.

[CIT0002] Alonso, V., L. M. Najes, L. Provincial, E. Guillén, M. Gil, P. Roncalés, and J. A. Beltrán. 2012. Influence of dietary fat on pork eating quality. Meat Sci. 92:366–373. doi:10.1016/j.meatsci.2012.01.004 22771111

[CIT0003] Ampuero Kragten, S., M. Collomb, S. Dubois, and P. Stoll. 2014. Composition des acides gras dans l’alimentation animale–méthodes d’analyse. Rech. Agron. Suisse 5:330–337.

[CIT0005] Bee, G., S. Gebert, and R. Messikommer. 2002. Effect of dietary energy supply and fat source on the fatty acid pattern of adipose and lean tissues and lipogenesis in the pig. J. Anim. Sci. 80:1564–1574. doi:10.2527/2002.8061564x 12078738

[CIT0006] Bee, G., A. L. Anderson, S. M. Lonergan, and E. Huff-Lonergan. 2007. Rate and extent of pH decline affect proteolysis of cytoskeletal proteins and water-holding capacity in pork. Meat Sci. 76:359–365. doi:10.1016/j.meatsci.2006.12.004 22064307

[CIT0007] Bee, G., S. Jacot, G. Guex, and C. Biolley. 2008. Effects of two supplementation levels of linseed combined with CLA or tallow on meat quality traits and fatty acid profile of adipose and different muscle tissues in slaughter pigs. Animal 2:800–811. doi:10.1017/S175173110800181X 22443606

[CIT0008] Bee, G., P. Silacci, S. Ampuero-Kragten, M. Čandek-Potokar, A. Wealleans, J. Litten-Brown, J. -P. Salminen, and I. Mueller-Harvey. 2017. Hydrolysable tannin-based diet rich in gallotannins has a minimal impact on pig performance but significantly reduces salivary and bulbourethral gland size. Animal 11:1617–1625. doi:10.1017/s1751731116002597 28004617 PMC5561437

[CIT0009] Berard, J., M. Kreuzer, and G. Bee. 2008. Effect of litter size and birth weight on growth, carcass and pork quality, and their relationship to postmortem proteolysis. J. Anim. Sci. 86:2357–2368. doi:10.2527/jas.2008-0893 18469061

[CIT0010] Berger, A., and J. B. German. 1990. Phospholipid fatty acid composition of various mouse tissues after feeding α-linolenate (18: 3n− 3) or eicosatrienoate (20: 3n− 3). Lipids 25:473–480. doi:10.1007/BF02538091 1977067

[CIT0011] Biondi, L., G. Luciano, D. Cutello, A. Natalello, S. Mattioli, A. Priolo, M. Lanza, L. Morbidini, A. Gallo, and B. Valenti. 2020. Meat quality from pigs fed tomato processing waste. Meat Sci. 159:107940. doi:10.1016/j.meatsci.2019.107940 31522104

[CIT0012] Cannata, S., T. E. Engle, S. J. Moeller, H. N. Zerby, A. E. Radunz, M. D. Green, P. D. Bass, and K. E. Belk. 2010. Effect of visual marbling on sensory properties and quality traits of pork loin. Meat Sci. 85:428–434. doi:10.1016/j.meatsci.2010.02.011 20416803

[CIT0013] Duan, Y., F. Li, L. Li, J. Fan, X. Sun, and Y. Yin. 2014. n-6: n-3 PUFA ratio is involved in regulating lipid metabolism and inflammation in pigs. Br. J. Nutr. 111:445–451. doi:10.1017/S0007114513002584 23947577

[CIT0014] Dugan, M. E., P. Vahmani, T. D. Turner, C. Mapiye, M. Juárez, N. Prieto, A. D. Beaulieu, R. T. Zijlstra, J. F. Patience, and J. L. Aalhus. 2015. Pork as a source of omega-3 (n-3) fatty acids. J. Clin. Med. 4:1999–2011. doi:10.3390/jcm4121956 26694475 PMC4693156

[CIT0015] European Commission. 2018. Directive (Eu) 2018/851 of the European Parliament and of the Council of 30 May 2018 amending Directive 2008/98/EC on waste. Off. J. Eur. Union 150:109–140.

[CIT0016] Ewaoluwagbemiga, E. O., G. Bee, and C. Kasper. 2023. Genetic analysis of protein efficiency and its association with performance and meat quality traits under a protein-restricted diet. Genet. Sel. Evol. 55:1–16. doi:10.1186/s12711-023-00812-3 37268880 PMC10236592

[CIT0017] Flowers, M. T., and J. M. Ntambi. 2009. Stearoyl-CoA desaturase and its relation to high-carbohydrate diets and obesity. Biochim. Biophys. Acta 1791:85–91. doi:10.1016/j.bbalip.2008.12.011 19166967 PMC2649790

[CIT0018] Font-i-Furnols, M., N. Tous, E. Esteve-Garcia, and M. Gispert. 2012. Do all the consumers accept marbling in the same way? The relationship between eating and visual acceptability of pork with different intramuscular fat content. Meat Sci. 91:448–453. doi:10.1016/j.meatsci.2012.02.030 22429803

[CIT0019] Gonzalez-Soto, M., and D. M. Mutch. 2021. Diet regulation of long-chain PUFA synthesis: role of macronutrients, micronutrients, and polyphenols on Δ-5/Δ-6 desaturases and elongases 2/5. Adv. Nutr. (Bethesda, Md.) 12:980–994. doi:10.1093/advances/nmaa142 PMC8166571 33186986

[CIT0020] Gutiérrez-Luna, K., I. Astiasarán, and D. Ansorena. 2022. Gels as fat replacers in bakery products: a review. Crit. Rev. Food Sci. Nutr. 62:3768–3781. doi:10.1080/10408398.2020.1869693 33412906

[CIT0021] Hibbeln, J. R., L. R. Nieminen, T. L. Blasbalg, J. A. Riggs, and W. E. Lands. 2006. Healthy intakes of n− 3 and n–6 fatty acids: estimations considering worldwide diversity. Am. J. Clin. Nutr. 83:1483S–1493S. doi:10.1093/ajcn/83.6.1483S 16841858

[CIT0022] Hoa, V. B., K. H. Seol, H. W. Seo, P. N. Seong, S. M. Kang, Y. S. Kim, S. S. Moon, J. H. Kim, and S. H. Cho. 2021. Meat quality characteristics of pork bellies in relation to fat level. Anim. Biosci. 34:1663–1673. doi:10.5713/ab.20.0612 33561922 PMC8495343

[CIT0023] Honikel, K. O. 1998. Reference methods for the assessment of physical characteristics of meat. Meat Sci. 49:447–457. doi:10.1016/s0309-1740(98)00034-5 22060626

[CIT0024] Hugo, A., and E. Roodt. 2007. Significance of porcine fat quality in meat technology: a review. Food Rev. Int. 23:175–198. doi:10.1080/87559120701225037

[CIT0025] ISO. 2008. ISO 16634-1 - Food products – Determination of the total nitrogen content by combustion according to the Dumas principle and calculation of the crude protein content – Part 1: oilseeds and animal feeding stuffs. Geneva (Switzerland): International Organisation for Standardisation.

[CIT0026] James, K., A. Millington, and N. Randall. 2022. Food and feed safety vulnerabilities in the circular economy. EFSA Support. Publ. 19:7226E. doi:10.2903/sp.efsa.2022.EN-7226

[CIT0027] Junior, D. T. V., I. B. Mandell, B. M. Bohrer, J. B. Dorleku, C. P. Campbell, T. E. Silva, E. Detmann, A. Saraiva, M. Juárez, and M. S. Duarte. 2024. Do carcass traits influence consumer perception of pork eating quality? Meat Sci. 208:109381. doi:10.1016/j.meatsci.2023.109381 37931578

[CIT0028] Kjos, N. P., M. Øverland, E. A. Bryhni, and O. Sørheim. 2000. Food waste products in diets for growing-finishing pigs: effect on growth performance, carcass characteristics and meat quality. Acta Agric. Scand. Sect. A 50:193–204. doi:10.1080/090647000750014322

[CIT0029] Kwak, W. S., and J. S. Kang. 2006. Effect of feeding food waste-broiler litter and bakery by-product mixture to pigs. Bioresour. Technol. 97:243–249. doi:10.1016/j.biortech.2005.02.008 16171681

[CIT0030] Lawless, H. T., and H. Heymann. 2010. Sensory evaluation of food: principles and practices. New York, NY: Springer Science & Business Media.

[CIT0031] Lawrie, R. A., and D. Ledward. 2014. Lawrie’s meat science. Cambridge, England: Woodhead Publishing.

[CIT0032] Lebret, B., and M. Čandek-Potokar. 2022. Pork quality attributes from farm to fork. Part I. Carcass and fresh meat. Animal 16:100402. doi:10.1016/j.animal.2021.100402 34836808

[CIT0033] Lee, H. C., A. Liang, Y. H. Lin, Y. R. Guo, and S. Y. Huang. 2018. Low dietary n-6/n-3 polyunsaturated fatty acid ratio prevents induced oral carcinoma in a hamster pouch model. Prostaglandins Leukot. Essent. Fatty Acids 136:67–75. doi:10.1016/j.plefa.2017.03.003 28292553

[CIT0034] Lenth, R. 2016. Least-Squares Means: The R package lsmeans. J. Stat. Softw. 61:1–33. doi:10.18637/jss.v069.i01.

[CIT0035] Liu, J., M. P. Ellies-Oury, T. Stoyanchev, and J. F. Hocquette. 2022. Consumer perception of beef quality and how to control, improve and predict it? Focus on eating quality. Foods 11:1732. doi:10.3390/foods11121732 35741930 PMC9223083

[CIT0036] López-Martínez, M. I., F. Toldrá, and L. Mora. 2023. Pork organs as a potential source of flavour-related substances. Food Res. Int. (Ottawa, Ont.) 173:113468. doi:10.1016/j.foodres.2023.113468 37803790

[CIT0037] Luciano, A., M. Tretola, S. Mazzoleni, N. Rovere, F. Fumagalli, L. Ferrari, M. Comi, M. Ottoboni, and L. Pinotti. 2021. Sweet vs. salty former food products in post-weaning piglets: effects on growth, apparent total tract digestibility and blood metabolites. Animals 11:3315. doi:10.3390/ani11113315 34828047 PMC8614654

[CIT0038] Luciano, A., C. D. Espinosa, L. Pinotti, and H. H. Stein. 2022. Standardized total tract digestibility of phosphorus in bakery meal fed to pigs and effects of bakery meal on growth performance of weanling pigs. Anim. Feed Sci. Technol. 284:115148. doi:10.1016/j.anifeedsci.2021.115148

[CIT0039] Martins, J. M., A. Albuquerque, J. A. Neves, A. B. Freitas, R. Charneca, and J. L. Tirapicos. 2018. Influence of outdoor rearing and oleic acid supplementation on lipid characteristics of muscle and adipose tissues from obese Alentejano pigs. J. Anim. Physiol. Anim. Nutr. (Berl) 102:e578–e590. doi:10.1111/jpn.12799 28990228

[CIT0040] Mazzoleni, S., M. Tretola, A. Luciano, P. Lin, L. Pinotti, and G. Bee. 2023. Sugary and salty former food products in pig diets affect energy and nutrient digestibility, feeding behaviour but not the growth performance and carcass composition. Animal 17:101019. doi:10.1016/j.animal.2023.101019 37967497

[CIT0041] Meilgaard, M. C., B. T. Carr, and G. V. Civille. 1999. Sensory evaluation techniques. Boca Raton (FL): CRC Press. doi:10.1201/9781003040729

[CIT0042] Minelli, G., K. D’Ambra, P. Macchioni, and D. P. Lo Fiego. 2023. Effects of pig dietary n-6/n-3 polyunsaturated fatty acids ratio and gender on carcass traits, fatty acid profiles, nutritional indices of lipid depots and oxidative stability of meat in medium–heavy pigs. Foods 12:4106. doi:10.3390/foods12224106 38002164 PMC10670070

[CIT0043] Mustonen, A. M., and P. Nieminen. 2023. Dihomo-γ-linolenic acid (20: 3n-6)—metabolism, derivatives, and potential significance in chronic inflammation. Int. J. Mol. Sci. 24:2116. doi:10.3390/ijms24032116 36768438 PMC9916522

[CIT0044] Nakamura, M. T., and T. Y. Nara. 2004. Structure, function, and dietary regulation of Δ6, Δ5, and Δ9 desaturases. Annu. Rev. Nutr. 24:345–376. doi:10.1146/annurev.nutr.24.121803.063211 15189125

[CIT0045] Navarro, M., F. R. Dunshea, A. Lisle, and E. Roura. 2021. Feeding a high oleic acid (C18: 1) diet improves pleasing flavor attributes in pork. Food Chem. 357:129770. doi:10.1016/j.foodchem.2021.129770 33866241

[CIT0046] Ngapo, T. M., L. Riendeau, C. Laberge, and J. Fortin. 2012. Marbling and ageing—Part 1. Sensory quality of pork. Food Res. Int. 49:396–405. doi:10.1016/j.foodres.2012.07.039

[CIT0047] Nguyen, L. Q., M. C. G. A. Nuijens, H. Everts, N. Salden, and A. C. Beynen. 2003. Mathematical relationships between the intake of n-6 and n-3 polyunsaturated fatty acids and their contents in adipose tissue of growing pigs. Meat Sci. 65:1399–1406. doi:10.1016/S0309-1740(03)00062-7 22063784

[CIT0048] Nieto, G., and G. Ros. 2012. Modification of fatty acid composition in meat through diet: effect on lipid peroxidation and relationship to nutritional quality—a review. Lipid Peroxidation 12:239–258. doi:10.5772/51114

[CIT0049] Nong, Q., L. Wang, Y. Zhou, Y. Sun, W. Chen, J. Xie, X. Zhu, and T. Shan. 2020. Low dietary n-6/n-3 PUFA ratio regulates meat quality, reduces triglyceride content, and improves fatty acid composition of meat in heigai pigs. Animals 10:1543. doi:10.3390/ani10091543 32882902 PMC7552283

[CIT0050] O’Connell, T. D., R. C. Block, S. P. Huang, and G. C. Shearer. 2017. ω3-Polyunsaturated fatty acids for heart failure: effects of dose on efficacy and novel signaling through free fatty acid receptor 4. J. Mol. Cell. Cardiol. 103:74–92. doi:10.1016/j.yjmcc.2016.12.003 27986444 PMC5944298

[CIT0051] Olivares, A., A. Daza, A. I. Rey, and C. J. Lopez-Bote. 2009. Interactions between genotype, dietary fat saturation and vitamin A concentration on intramuscular fat content and fatty acid composition in pigs. Meat Sci. 82:6–12. doi:10.1016/j.meatsci.2008.11.006 20416584

[CIT0052] Ospina-E, J. C., A. Sierra-C, O. Ochoa, J. A. Pérez-Álvarez, and J. Fernández-López. 2012. Substitution of saturated fat in processed meat products: a review. Crit. Rev. Food Sci. Nutr. 52:113–122. doi:10.1080/10408398.2010.493978 22059958

[CIT0053] Ottoboni, M., M. Tretola, A. Luciano, G. Giuberti, A. Gallo, and L. Pinotti. 2019. Carbohydrate digestion and predicted glycemic index of bakery/confectionary ex-food intended for pig nutrition. Ital. J. Anim. Sci. 18:838–849. doi:10.1080/1828051x.2019.1596758

[CIT0054] Pagliarini, E. 2021. Valutazione sensoriale: aspetti teorici, pratici e metodologici. Milan, Italy: HOEPLI EDITORE. p. 75–77

[CIT0055] Pinotti, L., A. Luciano, M. Ottoboni, M. Manoni, L. Ferrari, D. Marchis, and M. Tretola. 2021. Recycling food leftovers in feed as opportunity to increase the sustainability of livestock production. J. Clean Prod. 294:126290. doi:10.1016/j.jclepro.2021.126290

[CIT0056] Pinotti, L., L. Ferrari, F. Fumagalli, A. Luciano, M. Manoni, S. Mazzoleni, C. Govoni, M. C. Rulli, P. Lin, G. Bee, et al. 2023a. Pig-based bioconversion: the use of former food products to keep nutrients in the food chain. Animal 17:100918. doi:10.1016/j.animal.2023.100918 37544840

[CIT0057] Pinotti, L., S. Mazzoleni, A. Moradei, P. Lin, and A. Luciano. 2023b. Effects of alternative feed ingredients on red meat quality: a review of algae, insects, agro-industrial by-products and former food products. Ital. J. Anim. Sci. 22:695–710. doi:10.1080/1828051x.2023.2238784

[CIT0058] Rajeh, C., I. P. Saoud, S. Kharroubi, S. Naalbandian, and M. G. Abiad. 2021. Food loss and food waste recovery as animal feed: a systematic review. J. Mater. Cycles Waste Manage. 23:1–17. doi:10.1007/s10163-020-01102-6

[CIT0059] Saikia, A., G. Mejicanos, J. Rothy, E. Rajendiran, C. Yang, M. Nyachoti, H. Lei, R. Bergsma, Y. Wu, S. Jin, et al. 2024. Pork carcass composition, meat and belly qualities as influenced by feed efficiency selection in replacement boars from Large White sire and dam lines. Meat Sci. 210:109423. doi:10.1016/j.meatsci.2023.109423. http://hdl.handle.net/1993/37492 38218007

[CIT0060] Sandström, V., A. Chrysafi, M. Lamminen, M. Troell, M. Jalava, J. Piipponen, S. Siebert, O. van Hal, V. Virkki, and M. Kummu. 2022. Food system by-products upcycled in livestock and aquaculture feeds can increase global food supply. Nat. Food 3:729–740. doi:10.1038/s43016-022-00589-6 37118146

[CIT0061] Sinclair, A. J., N. M. Attar‐Bashi, and D. Li. 2002. What is the role of α‐linolenic acid for mammals? Lipids 37:1113–1123. doi:10.1007/s11745-002-1008-x 12617463

[CIT0062] Tikk, K., M. Tikk, M. D. Aaslyng, A. H. Karlsson, G. Lindahl, and H. J. Andersen. 2007. Significance of fat supplemented diets on pork quality–Connections between specific fatty acids and sensory attributes of pork. Meat Sci. 77:275–286. doi:10.1016/j.meatsci.2007.03.019 22061601

[CIT0063] Tretola, M., M. Ottoboni, A. Luciano, L. Rossi, A. Baldi, and L. Pinotti. 2019a. Former food products have no detrimental effects on diet digestibility, growth performance and selected plasma variables in post-weaning piglets. Ital. J. Anim. Sci. 18:987–996. doi:10.1080/1828051x.2019.1607784

[CIT0064] Tretola, M., F. Maghin, P. Silacci, S. Ampuero, and G. Bee. 2019b. Effect of supplementing hydrolysable tannins to a grower–finisher diet containing divergent PUFA levels on growth performance, boar taint levels in back fat and intestinal microbiota of entire males. Animals 9:1063. doi:10.3390/ani9121063 31810259 PMC6940899

[CIT0065] Van Laack, R. L. J. M., S. G. Stevens, and K. J. Stalder. 2001. The influence of ultimate pH and intramuscular fat content on pork tenderness and tenderization. J. Anim. Sci. 79:392–397. doi:10.2527/2001.792392x 11219448

[CIT0066] Wood, J. D., R. I. Richardson, G. R. Nute, A. V. Fisher, M. M. Campo, E. Kasapidou, P. R. Sheard, and M. Enser. 2004. Effects of fatty acids on meat quality: a review. Meat Sci. 66:21–32. doi:10.1016/S0309-1740(03)00022-6 22063928

[CIT0067] Wood, J. D., M. Enser, A. V. Fisher, G. R. Nute, P. R. Sheard, R. I. Richardson, S. I. Hughes, and F. M. Whittington. 2008. Fat deposition, fatty acid composition and meat quality: a review. Meat Sci. 78:343–358. doi:10.1016/j.meatsci.2007.07.019 22062452

[CIT0068] Yi, W., Q. Huang, Y. Wang, and T. Shan. 2023. Lipo-nutritional quality of pork: the lipid composition, regulation, and molecular mechanisms of fatty acid deposition. Anim. Nutr. 13:373–385. doi:10.1016/j.aninu.2023.03.001 37214214 PMC10196340

