# Supplementary material for: Sustainable pig diets: partial grain replacement with former food products and its impact on meat quality
Source: J Anim Sci. 2024 Mar 15;102:skae070. doi: 10.1093/jas/skae070 (PMC10989651; doi:10.1093/jas/skae070)
Supplement: skae070_suppl_Supplementary_Table_1 [file skae070_suppl_supplementary_table_1.docx]

Sustainable Pig Diets: Partial Grain Replacement with Former Food Products and its impact on Meat Quality

**Supplementary Table 1**

Analyzed composition (g/100g or MJ/kg on DM) of the two pure former food products used to formulate the experimental diets for growing-finishing pigs, similar to the two pure FFPs used for the diets in post-weaned piglets by Luciano et al. 2020.

| **Item** | **Pure SU FFPs^1^** | **Pure SA FFPs^2^** |
| --- | --- | --- |
| DM | 91.00 | 87.70 |
| DE (MJ/kg) | 19.60 | 19.40 |
| CP | 10.00 | 11.00 |
| Ash | 2.10 | 2.10 |
| Crude Fats (after hydrolysis) | 9.59 | 7.50 |
| CF | 1.60 | 2.20 |
| Starch | 42.50 | 50.50 |
| NFE | 67.80 | 64.90 |
| TS (expressed in sucrose) | 21.00 | 10.50 |
| Fe (mg/kg) | 41.70 | 95.00 |
| Sodium chloride | 0.20 | 0.15 |
| *Amino acids* |  |  |
| Arg | 0.48 | 0.20 |
| His | 0.19 | 0.17 |
| Ile | 0.33 | 0.27 |
| Leu | 0.59 | 0.68 |
| Lys | 0.26 | 0.18 |
| Met | 0.05 | 0.13 |
| Phe | 0.40 | 0.50 |
| Thr | 0.25 | 0.31 |
| Val | 0.40 | 0.27 |
| Ala | 0.29 | 0.66 |
| Asp | 0.48 | 0.40 |
| Cys | 0.10 | 0.10 |
| Glu | 2.44 | 2.87 |
| Gly | 0.32 | 0.48 |
| Pro | 0.80 | 1.34 |
| Ser | 0.40 | 0.54 |
| Tyr | 0.22 | 0.19 |
| Total | 8.00 | 9.29 |

Abbreviations: DE= digestible energy; CF= crude fiber; NFE= nitrogen-free extracts; TS= total sugars.

^1^ Pure SU FFPs: Pure confectionary former food products.

^2^ Pure SA FFPs: Pure bakery former food products.
